# High throughput whole mitochondrial genome sequencing by two platforms of massively parallel sequencing

**DOI:** 10.1186/1471-2164-15-S2-P7

**Published:** 2014-04-02

**Authors:** Seung Bum Seo, Xiangpei Zeng, Mourad Assidi, Bobby LaRue, Jonathan King, Antti Sajantila, Bruce Budowle

**Affiliations:** 1 Institute of Applied Genetics, Department of Forensic and Investigative Genetics, University of North Texas Health Science Centre, 3500 Camp Bowie Blvd., FoCentreth, Texas 76107, USA; 2 Center of Excellence in Genomic Medicine Research, King Abdulaziz University, Jeddah, Saudi Arabia; 3 KACST Technology Innovation Centre for Personalized Medicine at King AbdulAziz University, Jeddah, Saudi Arabia; 4 Department of Forensic Medicine, Hjelt Institute, University of Helsinki, Helsinki, Finland

## Background

Mitochondrial DNA (mtDNA) is a valuable genetic biomarker that has been implicated as a prognostic/diagnostic indicator for a number of diseases [1-3] as well as for human identification where forensic biologic evidence contains too little or no nuclear DNA, such as a hair shaft without root or a fingernail, or where a sample from a distant maternal relative is the only possibility for comparison [4-6]. Sanger sequencing has been the gold standard method for mtDNA typing, but the methodology has limitations with throughput, scalability, speed, and resolution [7]. Massively parallel sequencing technology (MPS) provides platforms for more comprehensive coverage of the genome per sample analyzed than currently is possible with Sanger sequencing [8,9]. Moreover, a number of different samples, which can be distinguished by barcoding, may be sequenced simultaneously. Two of the available personal genome sequencers are the Ion Torrent Personal Genome Machine (PGM™) (LifeTechnologies, San Francisco, CA) and the MiSeq^TM^ (Illumina, Inc., San Diego, CA). The PGM exploits non-optical sequencing on CMOS integrated circuits by detecting small changes in pH, due to release of H^+^ during addition of a nucleotide to the growing strand within a 2 hour run time. The MiSeq uses fluorescently tagged terminator chemistry and requires 39 hours for paired-end sequencing but has higher throughput and an associated simpler, less labor intensive library preparation methodology than the PGM.

With MPS potentially the mitochondrial genomes of 96 samples could be sequenced at one time. Sequencing of the entire mitochondrial genome provides higher resolution and discrimination power than is currently possible with only sequencing portions of the non-coding region of the mitochondrial genome (for high information content) or by targeted analyses (for a few SNP or deletions noted in the coding region).

## Materials and methods

**Figure 1:**
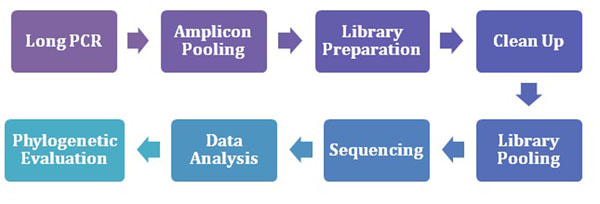
Basic MPS workflow for mtDNA sequencing.

DNA was extracted (QIAamp DNA Blood Mini Kit, Qiagen, Hilden, Germany) from whole blood of volunteers with informed consent. Whole genome mtDNA was amplified using primers that generate two amplicons approximately ~8.5 kb in length [9]. The general workflow is displayed in Figure 1.

The protocol for whole mitochondrial genome sequencing on the PGM is described on ion community (http://ioncommunity.lifetechnologies.com/community/applications/hid/mito/how_to). Ion Xpress™ Plus gDNA Fragment Library Kit, OneTouch™ 200 Template Kit v2 and Ion PGM™ 200 Sequencing Kit were mainly used for the library preparation, template preparation and sequencing reactions, respectively. On the MiSeq, the amplified DNA was fragmented and tagged using the NexteraXT protocol, indexed, size selected, and pooled for sequencing using the small amplicon targeted resequencing run, which performs 251bp paired end sequencing reads, according to the manufacturer’s recommendations

(http://supportres.illumina.com/documents/myillumina/900851dc-01cf-4b70-9e95-d590531c5bd4/nextera_xt_sample_preparation_guide_15031942_c.pdf); (http://support.illumina.com/sequencing/sequencing_instruments/miseq/training.ilmn) analyzed with the PGM platform and Ion 314™ Chip and 48 samples were analyzed on the MiSeq. Therefore, 23 samples were in common between the two MPS systems. In this study, sequencing of DNA was assessed for throughput, coverage, concordance of results between platforms, resolution of heteroplasmy and interpretation of homopolymeric stretch regions.

## Results

**Figure 2:**
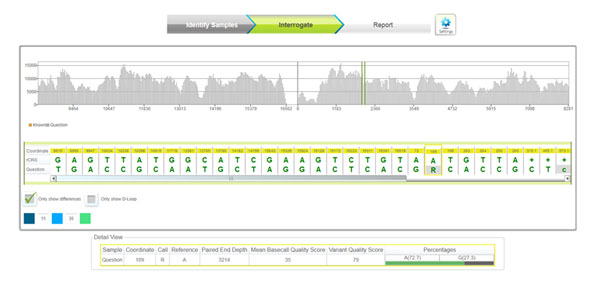
Coverage plot using MiSeq reporter displaying coverage and sequences of a selected region of two samples.

Average coverage of the 23 samples was at least 490X in the reactions (Figure 2):

Most variants were concordant between two different MPS platforms and with non-coding region data generated by Sanger sequencing. High quality phylogenetic scores (>89%) were obtained for the typing results from all samples with Haplogrep [10] (http://haplogrep.uibk.ac.at). While final calls were corrected manually, some regions were problematic. Most of these problematic areas were located at homopolymer regions due to base position shift and heteroplasmy. In addition, read strand bias was observed at several locations. Many of the discordant results can be corrected by software improvements.

## Conclusions

Overall, the PGM and MiSeq approaches generated good quality sequence data rapidly with relatively high coverage. The progress on these studies will be presented to provide insight on the near term applications and long term potential utility of MPS for both prognostic and diagnostic applications.
